# A comparative study on the location of the mandibular foramen in CBCT of normal occlusion and skeletal class II and III malocclusion

**DOI:** 10.1186/s40902-015-0024-2

**Published:** 2015-08-19

**Authors:** Hae-Seo Park, Jae-Hoon Lee

**Affiliations:** Department of Oral and Maxillofacial Surgery, College of Dentistry, Dankook University, 119 Dandae-ro, Dongnam-gu, Cheonan 330-714 Korea

## Abstract

**Background:**

During the orthognathic surgery, it is important to know the exact anatomical location of the mandibular foramen to achieve successful anesthesia of inferior alveolar nerve and to prevent damage to the nerves and vessels supplying the mandible.

**Methods:**

Cone-beam computed tomography (CBCT) was used to determine the location of the mandibular foramen in 100 patients: 30 patients with normal occlusion (13 men, 17 women), 40 patients with skeletal class II malocclusion (15 men, 25 women), 30 patients with skeletal class III malocclusion (17 men, 13 women).

**Results:**

The distance from the anterior border of the mandibular ramus to mandibular foramen did not differ significantly among the three groups, but in the group with skeletal class III malocclusion, this distance was an average of 1.43 ± 1.95 mm longer in the men than in the women (*p* < 0.05). In the skeletal class III malocclusion group, the mandibular foramen was higher than in the other two groups and was an average of 1.85 ± 3.23 mm higher in the men than in the women for all three groups combined (*p* < 0.05). The diameter of the ramus did not differ significantly among the three groups but was an average of 1.03 ± 2.58 mm wider in the men than in the women for all three groups combined (*p* < 0.05). In the skeletal class III malocclusion group, the ramus was longer than in the other groups and was an average of 7.9 ± 3.66 mm longer in the men than women.

**Conclusions:**

The location of the mandibular foramen was higher in the skeletal class III malocclusion group than in the other two groups, possibly because the ramus itself was longer in this group. This information should improve the success rate for inferior alveolar nerve anesthesia and decrease the complications that attend orthognathic surgery.

## Background

The mandibular foramen is located inside the mandibular ramus and serves as a passageway for blood vessels that supply nutrients to the mandible, mandibular teeth, periodontal tissues, and lower lip and for the nerves responsible for sensory perception in these regions. Thus, locating the accurate anatomical position of these regions is critical to achieving more successful inferior alveolar nerve block and preventing the complications common to orthognathic surgery [1].

Inferior alveolar nerve block anesthesia is intended to reduce pain during surgery (e.g., for tooth extractions and implantations in the mandible). The anesthetic is delivered right above the mandibular foramen, and the effect is often inadequate if the surgeon does not accurately identify the anatomical site of the foramen or if it has been dislocated [2]. In addition, orthognathic surgery intended to correct maxillofacial deformities or for aesthetic reasons may result in complications (e.g., damage to inferior alveolar nerve and local blood vessels) unless the foramen can be located precisely [3].

Radiographic images are obtained clinically to identify the position of the mandibular foramen. Although the panoramic view can be used for this purpose, it has the disadvantage of being less accurate owing to phase transformations and magnification. Computed tomography (CT) can accurately identify the position of the foramen three-dimensionally but is expensive and exposes the patient to an excessive dose of radiation. The recent introduction of cone-beam computed tomography (CBCT) has overcome such disadvantages, acquiring images through a one-time rotation [4, 5] at a lower cost, a lower dose and with easier operation, as compared with conventional multislice CT [4, 6, 7].

Many studies have been carried out to determine the best method for locating the mandibular foramen. Alves et al. [8] analyzed its anatomical position by measuring the mandible (in 185 cases), and da Fontoura et al. [9] determined its position in dry mandibles (in 140 cases) and compared the findings with those obtained on panoramic views. Trost et al. [10] examined the mandible (in 46 cases) to determine the relative positions of the mandibular ramus and the mandibular foramen, and Seo et al. [11] used panoramic radiography to compare differences in the position of the mandibular foramen between patients with normal occlusion and those with mandibular prognathism.

Nevertheless, few studies have compared the anatomical position of the mandibular foramen in patients with skeletal class II and class III malocclusions for whom orthognathic surgery is often performed. In this study, CBCT, which is now in wide clinical use, was chosen to compare the anterior-posterior position and vertical positions of the mandibular foramen among patients with normal occlusion and those with skeletal class II or III malocclusions.

## Methods

### Study subjects

The study included 100 patients who visited the Dankook University Dental Hospital from January 2013 to June 2014. Cephalometric analysis provided A point-nasion-B point (ANB) values that were used to classify the patients into three groups: 30 with normal occlusion, 40 with skeletal class II malocclusion, and 30 with skeletal class III malocclusion. Subjects was 18 to 31 years of age as of the dates when the CBCT radiographs were obtained. Included in the skeletal class II group were 30 patients in division 1 and 10 patients in division 2 of this class. Patients who underwent orthognathic surgery and had facial asymmetry were excluded.

### Study methods

#### CBCT

CBCT radiographs taken using PHT-60FO (VATECH Corp., Hwa-Sung, Korea) were reconstructed in a panoramic view using Invivo 5.1 (Anatomage, San Jose, CA, USA). Analysis of the mandibular foramen was on the region where the mandibular canal ended.

#### CBCT analysis

**Fig. 1:**
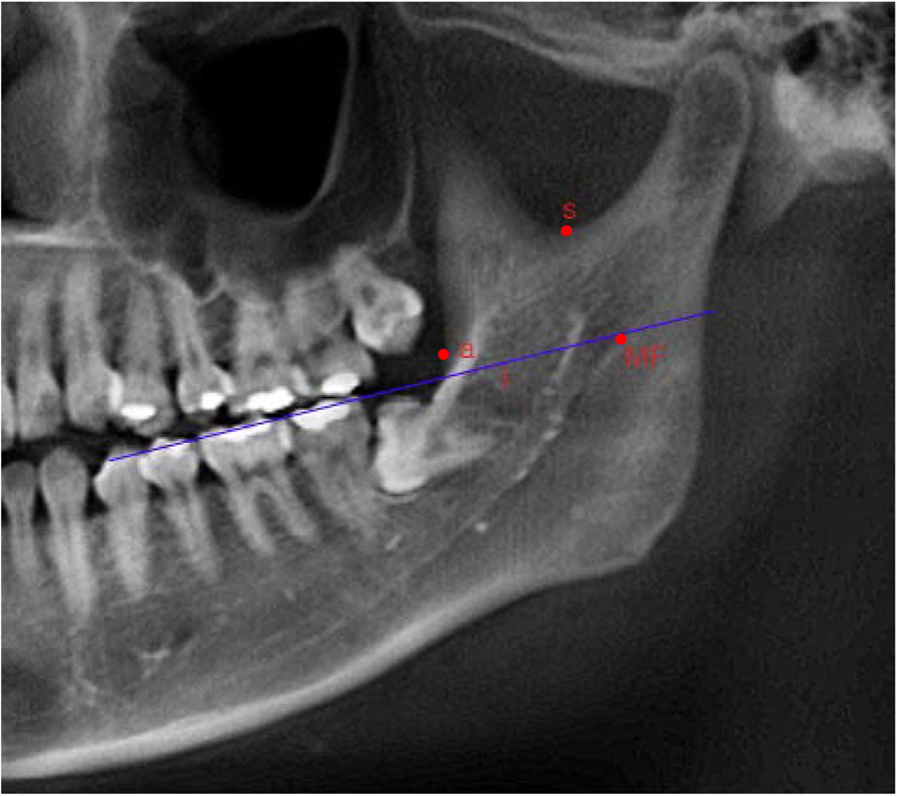
Reference point and line

The mandibular foramen (MF) was set posterosuperior to the opening of mandibular canal. The deepest point of the anterior edge of the mandibular ramus was named ‘a’ and the lowest point of mandibular notch was named ‘s’. The extension of the occlusal plane connecting the mesio-occlusal line angle of the first premolars and the postero-occlusal line angle of the second molars was given a value of ‘l’ and used as a reference in comparing the positions of the mandibular foramen (Table 1, Fig. 1).

**Table 1:** Reference point and line

MF	Posteriorsuperior point of mandibular canal opening
a	Deepest point on anterior border of ascending ramus
s	Lowest point of mandibular notch
l	Extension line of occlusal plane

**Fig. 2:**
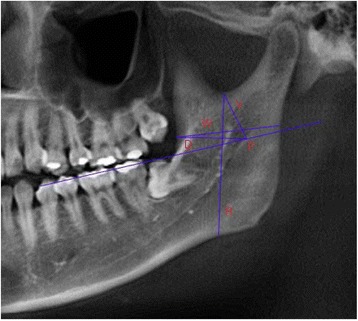
Items of measurement

To compare the anterior and posterior positions of mandibular foramen, the distance (D, in mm) between ‘a’ and the MF and the shortest distance (W, in mm) between ‘a’ and the posterior border of mandibular ramus were measured. To compare the vertical position of the mandibular foramen, the length (V, in mm) between ‘s’ and the MF and the length of the perpendicular line (P, in mm) from the extension of occlusal plane to the MF and the shortest distance (R, in mm) between ‘s’ and the inferior edge of the mandible was measured (Table 2, Fig. 2).

**Table 2:** Items of measurement

W	Diameter of ramus
D	Distance of MF from a
V	Distance of MF from s
P	Distance of MF from l
R	Distance of Mandibular lower border from s

#### Evaluation techniques

In CBCT images reconstructed in a panoramic view, items were measured in the mandibular ramus on both sides. First, the measurements were made in the three groups of patients (those with normal occlusion, those with skeletal class II malocclusion, and those with skeletal class III malocclusion). Next, same-gender comparisons were made among the three groups. Men and women within the same group were compared, followed by a comparison between male and female patients in the entire cohort. Finally, within the skeletal class II malocclusion group, the two subsets of patients in division 1 and division 2 were compared.

#### Statistical analysis

When the subject of a comparison was included in all three groups, a one-way analysis of variance (ANOVA) test was conducted, followed by the Tukey test to verify the results. When the subject of a comparison included in two of the patient groups, an independent sample *t*-test was performed. Differences were considered statistically significant if the *p* value was less than 0.05.

## Results

CBCT images of the patients in the three study groups (normal occlusion, skeletal class II malocclusion, and skeletal class III malocclusion) were compared with respect to the position of the mandibular foramen. The findings are summarized as follows.

### Patient groups

Of the 100 patients, 45 patients were men and 55 were women, with a mean age of 21.5 ± 3.2 years. Of the 30 patients with normal occlusion, 13 were men and 17 were women, with a mean age was 21.8 ± 3.4 years. The average sella-nasion-A point angle (SNA) was 83.05°, the average sella-nasion-B point angle (SNB) was 80.22°, and the ANB averaged 2.83°. Of the 40 patients with skeletal class II malocclusion, 15 were men and 25 were women, with a mean age of 22.2 ± 3.0 years. The average SNA was 82.42°, the average SNB was 76.38°, and the average ANB was 6.04°. Of the 30 patients with skeletal class III malocclusion, 17 were men and 13 were women, with a mean age 20.7 ± 3.2 years. The average SNA was 81.42°, the average SNB was 82.53°, and the average ANB was −1.12° (Table 3).

**Table 3:** Sex, age of each group

Group	Sex		Age	SNA	SNB	ANB
A	Men: 13	Women: 17	21.8 (18 ~ 31)	83.05°	80.22°	2.83°
B	Men: 15	Women: 25	22.2 (18 ~ 30)	82.42°	76.38°	6.04°
C	Men: 17	Women: 13	20.7 (18 ~ 31)	81.42°	82.53°	−1.12°

A: Normal occlusion group

B: Class II malocclusion group

C: Class III malocclusion group

### Inter-group comparisons

There were no significant difference in ‘D’, the anterior-posterior position of the mandibular foramen, and in ‘W’, the anterior-posterior width of the mandibular ramus among three groups. But there were significant difference in ‘V’ and ‘P’, the vertical position of the mandibular foramen (*p* <0.05). The average V measurements (s-MF) were 21.59 mm in the normal occlusion group, 20.49 mm in the skeletal class II malocclusion group, and 18.77 mm in the skeletal class III malocclusion group. The average P measurements (l-MF) were 0.10 mm below the occlusal plane in the normal occlusion group, 0.03 mm below the occlusal plane in the skeletal class II malocclusion group, and 2.79 mm higher the occlusal plane in the skeletal class III malocclusion group. In addition, the average values of R (s-mandible lower border), the length of the mandibular ramus, differed significantly among the three groups (*p* <0.05): 48.38 mm in the normal occlusion group, 44.47 mm in the skeletal class II malocclusion group, and 52.94 mm in the skeletal class III malocclusion group. These results indicate progressive increases in the length of mandibular ramus from the skeletal class II malocclusion group to the normal occlusion group to the skeletal class III malocclusion group (Table 4).

**Table 4:** Comparison of normal occlusion, skeletal class II and III malocclusion group

	Group	Mean	SD	Significance		
				A-B	A-C	B-C
W	A	31.6615 mm	±2.75349			
	B	31.6040 mm	±2.19749			
	C	31.1930 mm	±2.87840			
D	A	19.4077 mm	±2.15708			
	B	19.0080 mm	±1.82927			
	C	19.8488 mm	±2.03664			
V	A	21.5887 mm	±2.45216		*	*
	B	20.4897 mm	±2.86414			
	C	18.7697 mm	±2.40634			
P	A	−0.0980 mm	±2.97157		*	*
	B	−0.0333 mm	±3.02146			
	C	2.7877 mm	±2.50825			
R	A	48.3750 mm	±5.77211	*	*	*
	B	44.4713 mm	±4.44070			
	C	52.9430 mm	±5.78652			

A: Normal occlusion group

B: Class II malocclusion group

C: Class III malocclusion group

* significant at the level of *P* < 0.05

### Gender comparisons within each group

#### Comparisons between male patients for each group

There were no significant difference in ‘D’, the anterior-posterior position of the mandibular foramen, and in ‘W’, the anterior and posterior width of the mandibular ramus among three groups. But there were significant difference in ‘V’ and ‘P’, the vertical position of the mandibular foramen (*p* <0.05). With regard to the average V measurements (s-MF), there was a significant difference between the normal occlusion group and the other two groups: 22.29 mm in the normal occlusion group, 19.70 mm in the skeletal class II malocclusion group, and 19.62 mm in the skeletal class III malocclusion group. With regard to the average P measurements (l-MF), there was a significant difference between the skeletal class III malocclusion group and the other two groups: 0.73 mm above the occlusal plane in the normal occlusion group, 0.70 mm above the occlusal plane in the skeletal class II malocclusion group, and 3.56 mm above the occlusal plane in the skeletal class III malocclusion group. In addition, with regard to the average R measurements (s-mandible lower border), the length of the mandibular ramus was 53.27 mm in the normal occlusion group, 47.02 mm in the skeletal class II malocclusion group, and 56.89 mm in the skeletal class III malocclusion group. The difference between the skeletal class II and III malocclusion groups was significant, and the length of mandibular ramus in the skeletal class III malocclusion group was relatively longer than in the other two groups (Table 5).

**Table 5:** Comparison of normal occlusion, skeletal class II and III malocclusion group (only men)

	Group	Mean	SD	Significance		
				A-B	A-C	B-C
W	A	31.9108 mm	±2.94028			
	B	32.3123 mm	±2.42030			
	C	31.9850 mm	±2.44729			
D	A	19.2100 mm	±2.35720			
	B	19.1659 mm	±1.91019			
	C	20.4659 mm	±1.95319			
V	A	22.2931 mm	±2.36451	*	*	
	B	19.6991 mm	±3.63638			
	C	19.6232 mm	±2.32103			
P	A	0.7277 mm	±3.05969		*	*
	B	0.7000 mm	±3.46783			
	C	3.5553 mm	±2.46164			
R	A	53.2700 mm	±5.72731			*
	B	47.0150 mm	±5.06719			
	C	56.8920 mm	±3.65576			

A: Normal occlusion group

B: Class II malocclusion group

C: Class III malocclusion group

* significant at the level of *P* < 0.05

#### Comparisons between female patients for each group

There were no significant difference in ‘D’, the anterior-posterior position of the mandibular foramen, and in ‘W’, the anterior and posterior width of the mandibular ramus. However, there were significant differences in ‘V’ and ‘P’, the vertical position of the mandibular foramen, in the skeletal class III malocclusion group and the other two groups (*p* <0.05). In the average V measurements (s-MF) were 21.05 mm in the normal occlusion group, 20.95 mm in the skeletal class II malocclusion group, and 18.31 mm in the skeletal class III malocclusion group. In the average P measurements (l-MF) were 0.73 mm below the occlusal plane in the normal occlusion group, 0.46 mm below the occlusal plane in the skeletal class II malocclusion group, and 1.78 mm above the occlusal plane in the skeletal class III malocclusion group. In addition, with regard to the average R measurements (s-mandible lower border), the length of the mandibular ramus was 47.15 mm in the normal occlusion group, 43.55 mm in the skeletal class II malocclusion group, and 48.99 mm in the skeletal class III malocclusion group. The difference was significant only between the skeletal class II and III malocclusion groups, and the length of the mandibular ramus was relatively longer in the skeletal class III malocclusion group (Table 6).

**Table 6:** Comparison of normal occlusion, skeletal class II and III malocclusion group (only women)

	Group	Mean	SD	Significance		
				A-B	A-C	B-C
W	A	31.4709 mm	±2.63046			
	B	31.1939 mm	±1.97599			
	C	30.1573 mm	±3.11106			
D	A	19.5588 mm	±2.01378			
	B	18.9166 mm	±1.80043			
	C	19.0419 mm	±1.88635			
V	A	21.0500 mm	±2.41356		*	*
	B	20.9474 mm	±2.23361			
	C	18.3073 mm	±2.48235			
P	A	−0.7294 mm	±2.78340		*	*
	B	−0.4579 mm	±2.68832			
	C	1.7838 mm	±2.23594			
R	A	47.1513 mm	±5.25874			*
	B	43.5464 mm	±3.91307			
	C	48.9940 mm	±4.76117			

A: Normal occlusion group

B: Class II malocclusion group

C: Class III malocclusion group

* significant at the level of *P* < 0.05

### Comparisons between men and women in the same group

There was no significant differences in the anterior-posterior or vertical positions of the mandibular foramen, the anterior-posterior width of ramus, or the vertical length of the ramus in the normal occlusion group and in the skeletal class II malocclusion group (Tables 7, 8). However, in the skeletal class III malocclusion group, there were significant differences between the men and women in the average values of D (the anterior-posterior position of the mandibular foramen), W (the anterior-posterior width of the mandibular ramus), P (the vertical position of the mandibular foramen), and R (the length of the mandibular ramus) (*p* <0.05). The average D (a-MF) measured 20.47 mm in the men and 19.04 mm in the women. The average W (a-mandible posterior border) was 31.99 mm in the men and 30.16 mm in the women. The average P (l-MF) was 3.56 mm above the occlusal plane in the men and 1.78 mm above the occlusal plane in the women. The average R (s-mandible lower border) was 56.89 mm in the men and 48.99 mm in the women (Table 9).

**Table 7:** Comparison of men and women (in normal occlusion group)

	Group	Mean	SD	Significance
W	Men	31.9108 mm	±2.94028	
	Women	31.4709 mm	±2.63046	
D	Men	19.2100 mm	±2.35720	
	Women	19.5588 mm	±2.01378	
V	Men	22.2931 mm	±2.36451	
	Women	21.0500 mm	±2.41356	
P	Men	0.7277 mm	±3.05969	
	Women	−0.7294 mm	±2.78340	
R	Men	53.2700 mm	±5.72731	
	Women	47.1513 mm	±5.25874	

* significant at the level of *P* < 0.05

**Table 8:** Comparison of men and women (in skeletal class II malocclusion group)

	Group	Mean	SD	Significance
W	Men	32.3123 mm	±2.42030	
	Women	31.1939 mm	±1.97599	
D	Men	19.1659 mm	±1.91019	
	Women	18.9166 mm	±1.80043	
V	Men	19.6991 mm	±3.63638	
	Women	20.9474 mm	±2.23361	
P	Men	0.7000 mm	±3.46783	
	Women	−0.4579 mm	±2.68832	
R	Men	47.0150 mm	±5.06719	
	Women	43.5464 mm	±3.91307	

* significant at the level of *P* < 0.05

**Table 9:** Comparison of men and women (in skeletal class III malocclusion group)

	Group	Mean	SD	Significance
W	Men	31.9850 mm	±2.44729	*
	Women	30.1573 mm	±3.11106	
D	Men	20.4659 mm	±1.95319	*
	Women	19.0419 mm	±1.88635	
V	Men	19.1232 mm	±2.32103	
	Women	18.3073 mm	±2.48235	
P	Men	3.5553 mm	±2.46164	*
	Women	1.7838 mm	±2.23594	
R	Men	56.8920 mm	±3.65576	*
	Women	48.9940 mm	±4.76117	

* significant at the level of *P* < 0.05

### Intergender comparisons within the total study group

There was no significance difference between the men and the women with respect to D (the anterior-posterior position of the mandibular foramen), yet there were significant differences in W (the anterior-posterior width of the mandibular ramus), P (the vertical position of the mandibular foramen), and R (the length of mandibular ramus) (*p* <0.05). The average W (a-mandible posterior border) was 32.05 mm in the men and 31.02 mm in the women. The average P (l-MF) was 1.89 mm above the occlusal plane in the men and 0.04 mm above the occlusal plane in the women. The average R (s-mandible lower border) was 52.64 mm in the men and 45.88 mm in the women (Table 10).

**Table 10:** Comparison of men and women

	Group	Mean	SD	Significance
W	Men	32.0493 mm	±2.57948	*
	Women	31.0150 mm	±2.57302	
D	Men	19.7189 mm	±2.14839	
	Women	19.1727 mm	±1.90151	
V	Men	20.2828 mm	±3.05004	
	Women	20.2826 mm	±2.62647	
P	Men	1.8927 mm	±3.23393	*
	Women	0.0427 mm	±2.79296	
R	Men	52.6418 mm	±6.30166	*
	Women	45.8829 mm	±5.01705	

* significant at the level of *P* < 0.05

### Comparison between division 1 and division 2 subsets of patients with skeletal class II malocclusion group

There were no significant differences between the division 1 and division 2 patients with respect to the anterior, posterior, or vertical positions of the mandibular foramen or the width and length of the mandibular ramus (Table 11).

**Table 11:** Comparision of division 1 and 2 (skeletal class II malocclusion)

	Group	Mean	SD	Significance
W	Division 1	31.6522 mm	±2.21051	
	Division 2	31.5075 mm	±2.22499	
D	Division 1	19.1013 mm	±1.89594	
	Division 2	18.8215 mm	±1.71978	
V	Division 1	19.9697 mm	±2.97195	
	Division 2	20.5795 mm	±2.52002	
P	Division 1	−0.1140 mm	±2.83028	
	Division 2	0.1280 mm	±2.22499	
R	Division 1	44.2195 mm	±4.35233	
	Division 2	44.9750 mm	±4.80920	

* significant at the level of *P* < 0.05

## Discussion

The mandibular foramen and the mandibular canal form during the process of intramembranous ossification of the mandibular ramus and the body of mandible. During the 24th week of the embryonic stage, a groove forms that contains the nerves and blood vessels, and the shapes of the mandibular foramen and canal completed as ossification progresses [12]. Starting from the mandibular foramen within the ramus, the mandibular canal containing the inferior alveolar nerve and blood vessels descends in antero-inferior direction and then runs horizontally once it reaches the molar area of the mandible body. At this point the canal splits into the incisive canal, which runs from the premolar to the anterior mandible, and the mental canal, which runs in a postero-superior direction and opens below the apical root of the second premolar, becoming the mental foramen [13].

Although inferior alveolar nerve block is frequently used as a local anesthetic method for restorative treatment and surgical treatment of mandibular molars [14, 15], Malamed et al. [16] reported that this method is associated with a high clinical failure rate of up to 15 to 20 %. This can be explained by the fact that the positions of the mandibular ramus and foramen vary widely from person to person [14]. In addition, if surgeon fails to identify the exact anatomical position of the mandibular foramen during orthognathic surgery, complications may ensue, such as damage to the inferior alveolar nerve or blood vessels. Thus, the position of the foramen serves as a critical anatomical reference point for reducing the risk of complications and for the success of inferior alveolar nerve block anesthesia [3].

The position of the mandibular foramen is known to vary with age. According to Hwang et al. [17], the mandibular foramen is located below the occlusal plane during the deciduous dentition stage and is positioned at 4.14 mm above the occlusal plane in adults. Kanno et al. [18] have reported that the mandibular lingula can be seen at 6 mm above the occlusal plane in children ages 7 to 8 and at 10 mm above the occlusal plane in children ages 9 to 10. In addition, during deciduous dentition the anterior-posterior position of the foramen is in center of the ramal surface but will then move slightly toward the back [19]. To avoid the possible effect of this positional change, in this study, only patients who were 18 to 31 years of age were included.

It is critically important to accurately identify the position of the mandibular foramen in the clinical setting. Radiography makes this possible in a non-invasive manner, and panoramic radiography is the most commonly used technique, because it enables the practitioner to simultaneously observe the teeth, jaw and temporomandibular joint [20–22]. Although panoramic radiography is cost-effective and easy to handle, magnification varies depending on camera type [1], which may result in distorted or deformed images if the patient’s jaw bone is not positioned correctly in the focal trough. CT enables a three-dimensional evaluation by the reconstruction of images, resulting in better precision. With the images obtained by means of panoramic radiography, the probability of identifying the incisive canal in the mandible in two different studies was 2.7 % [23] to about 15 % [20]. In comparison, three studies evaluated CT images and reported corresponding values to be 83 % [24] to 100 % [25, 26]. Although CT offers many advantages, it is expensive and difficult to use in the clinical setting and is therefore employed less often. In contrast, when compared with conventional CT, CBCT requires a lower dose of radiation, is easier to control, and minimizes metal artifact and is therefore used more frequently in clinical setting. For these reasons, this study relied on the more accessible CBCT to analyze the position of the mandibular foramen.

In their study comparing the anterior-posterior position of the mandibular foramen, da Fontoura et al. [9] noted that the foramen is positioned in the middle third of ramus. In addition, Trost et al. [10] found that the mandibular foramen did not exist in the region of the upper and posterior third of the ramus. In their study of the distance from the anterior edge of the ramus to the foramen in a horizontal relationship, Afsar et al. [27] analyzed radiographs and found this distance to be an average of 20.20 mm. In their analysis of 40 Taiwanese patients, Yu et al. [28] reported this distance to average 18.00 mm in the women and 19.30 mm in the men. In addition, Kaffe et al. [1] measured this distance as an average of 20.26 mm using panoramic radiography of dry mandibles.

In contrast, Seo et al. [11] in their examination of panoramic radiographs from Korean patients diagnosed with either normal occlusion or prognathism, compared the distance from the anterior edge of the ramus to the mandibular foramen and found it to be average of 24.48 mm in those with normal occlusion and 24.535 mm in those with prognathism. In this study using CBCT, however, this distance was an average of 19.41 mm, 19.01 mm, and 19.85 mm in the normal occlusion group, the skeletal class II malocclusion group, and the skeletal class III malocclusion group, respectively. Although these results were lower than those in the study by Seo et al. [11] and there was no significant difference among the three groups, when this distance compared in the male and female patients in the skeletal class III malocclusion group, it averaged 20.47 mm in the men and 19.04 mm in the women–that is, 1.43 mm longer in the men–and the difference was statistically significant (*p* <0.05). With regard to the horizontal width of ramus, the average values were 31.99 mm in the men and 30.16 mm in the women–that is, 1.83 mm longer in the men.

Lima et al. [29] compared the vertical position of the mandibular foramen and reported that the distance from mandibular notch to the foramen served as a critical reference point for identifying the position of the mandibular foramen during orthognathic surgery and thus gave it clinical importance. They reported an average distance of 27.70 mm, whereas Gutierrez-Ventura et al. [30] reported an average distance of 17.44 mm. In a report by Yu et al. [28], these values were 22.70 mm in their male patients and 20.50 mm in their female patients. In this study, the distance from the mandibular notch to the mandibular foramen was 21.59 mm in the normal occlusion group, 20.49 mm in the skeletal class II malocclusion group, and 18.77 mm in the skeletal class III malocclusion group. These values were similar to those reported in other studies, although the value was significantly lower in the skeletal class III malocclusion group.

In an analysis of the occlusal plane, the position of mandibular foramen was 0.10 mm below this plane in the normal occlusion group, 0.03 mm below this plane in the skeletal class II malocclusion group, and, significantly, 2.79 mm above the occlusal plane in the skeletal class III malocclusion group. These results were the same as those in a study conducted by Seo et al. [11].

The average vertical length of the mandibular ramus at about 2.89 mm above the occlusal plane. The average vertical length of the mandibular ramus was 48.38 mm in the normal occlusion group, 44.47 mm in the skeletal class II malocclusion group, and 52.94 mm in the skeletal class III malocclusion group, in whom it was the longest. This difference appears to be the reason for the difference in the vertical position of the mandibular foramen among the groups studied.

In addition, there were significant differences in the distance from the occlusal plane to the mandibular foramen in the intergender comparisons. Specifically, this distance averaged 1.89 and 0.04 mm in the men and women, respectively, or 1.85 mm greater in the male patients. The average vertical length of the ramus was 52.6 mm in the men and 45.88 mm in the women, or about 6.76 mm longer in the male patients. These results were consistent with those of a study by Indira et al. [31] and indicate that variations in the vertical length are due to differences in the length of the mandible ramus.

Until now, no other study has compared the position of the mandibular foramen among patients with normal occlusion, skeletal class II and III malocclusion. Here, CBCT images determined that the position of the foramen varies from person to person and is higher in patients with skeletal class III malocclusion. The intergender comparisons showed that the mandibular foramen is higher in men than in women. Thus, CBCT can most likely be used effectively to evaluate the position of the mandibular foramen prior to inferior alveolar nerve block anesthesia or orthognathic surgery. In addition, it will be useful to reconstruct images of the mandible obtained with CBCT to create three-dimensional images and then to compare the position of the mandibular foramen with the results of this study.

## Conclusions

In this study, the position of the mandibular foramen was determined with use of CBCT and compared in patients with normal occlusion, skeletal class II and III malocclusion. The following is a summary of the results.

The anterior-posterior position of the mandibular foramen, as based on the deepest point on the anterior edge of the ramus, averaged 19.41 mm (men, 19.21 mm; women, 19.59 mm) in the normal occlusion group. It averaged 19.01 mm in the skeletal class II malocclusion group (men, 19.17 mm; women, 18.92 mm) and 19.85 mm in the skeletal class III malocclusion group (men, 20.47 mm; women, 19.04 mm), indicating no significant difference among the three groups. This distance in men was 1.43 mm longer than in the women for the skeletal class III malocclusion group, a finding that was statistically significant (*p* <0.05).

The vertical position of the mandibular foramen, as based on the extension of the occlusal plane, averaged −0.10 mm in the normal occlusion group (men, 0.73 mm; women, −0.72 mm), −0.03 mm in the skeletal class II malocclusion group (men, 0.70 mm; women, −0.46 mm), and 2.79 mm in the skeletal class III malocclusion group (men, 3.56 mm; women, 1.78 mm), that is, it was higher (in both men and women) in the patients with skeletal class III malocclusion (*p* <0.05). In addition, the mandibular foramen was positioned approximately 1.85 mm higher in the men than in the women (*p* <0.05).

The full width of the mandibular ramus averaged 31.66 mm in the normal occlusion group (men, 31.91 mm; women, 31.47 mm), 31.60 mm in the skeletal class II malocclusion group (men, 32.31 mm; women, 31.19 mm), and 31.19 mm in the skeletal class III malocclusion group (men, 31.99 mm; women, 30.16 mm), indicating no significant difference between these three groups. This width was approximately 1.03 mm longer in the men than in the women, and the difference was statistically significant (*p* <0.05).

The average length of the mandibular ramus was 48.38 mm in the normal occlusion group (men, 53.27 mm; women, 47.15 mm), 44.47 mm in the skeletal class II malocclusion group (men, 47.02 mm; women, 43.55 mm), and 52.94 mm in the skeletal class III malocclusion group (men, 56.89 mm; women, 48.99 mm). Thus, this length was greatest in the skeletal class III malocclusion group (*p* <0.05) and was 7.9 mm longer in the men than in the women (*p* <0.05).

When the skeletal class II malocclusion group division 1 and division 2 patients were compared, there were no significant differences in these measurements.

In this study, CBCT, which is now in widespread use, was chosen as the best method for analyzing the positional relationship of the mandibular foramen to surrounding structures. According to the results reported here, the position of the mandibular foramen varies from person to person, and in the skeletal class III malocclusion patients it was located higher than the position in the other two groups. More likely, this is because the length of ramus in the skeletal class III malocclusion group exceeds that in the other two groups.
